# Development of a Dynamic Oriented Rehabilitative Integrated System (DORIS) and Preliminary Tests

**DOI:** 10.3390/s19153402

**Published:** 2019-08-02

**Authors:** Susanna Summa, Riccardo Gori, Luigi Freda, Enrico Castelli, Maurizio Petrarca

**Affiliations:** 1 MARlab, Neuroscience and Neurorehabilitation Department, Bambino Gesù Children’s Hospital—IRCCS, 00165 Rome, Italy; 2 Elemize Technologies s.r.l., 00153 Rome, Italy; 3 Inglobe Technologies s.r.l., 03023 Ceccano (FR), Italy

**Keywords:** posturography, stewart platform, rehabilitation

## Abstract

Moving platforms were introduced in the field of the study of posturography since the 1970s. Commercial platforms have some limits: a limited number of degrees of freedom, pre-configured protocols, and, usually, they are expensive. In order to overcome these limits, we developed a robotic platform: Dynamic Oriented Rehabilitative Integrated System (DORIS). We aimed at realizing a versatile solution that can be applied both for research purposes but also for personalizing the training of equilibrium and gait. We reached these goals by means of a Stewart platform that was realized with linear actuators and a supporting plate. Each actuator is provided by an ad hoc built monoaxial load cell. Position control allows a large range of movements and load cells measure the reactive force applied by the subject. Transmission Control Protocol/Internet Protocol (TCP/IP) guarantees the communication between the platform and other systems. We integrated DORIS with a motion analysis system, an electromyography (EMG) system, and a virtual reality environment (VR). This integration and the custom design of the platform offer the opportunity to manipulate the available information of the subject under analysis, which uses visual, vestibular, and plantar feet pressure inputs. The full access to the human movements and to the dynamic interaction is a further benefit for the identification of innovative solutions for research and physical rehabilitation purposes in a field that is widely investigated but still open.

## 1. Introduction

Nowadays, it is well established that, in the rehabilitative field, it is necessary to be up to date with the scientific research breakthroughs and to take into consideration motor control knowledge. In order to effectively introduce the rehabilitation in a scientific context, it is necessary to report observations in an objective and repeatable framework and it is necessary to conduct accurate measures under controlled conditions. The study of the movement is useful to characterize the strategies of motor control during actions like reaching, standing, or walking in normal or pathological conditions, and their modifications following cycles of therapy [1]. In this context, further knowledge and tools are needed to dose and to verify the efficacy of the therapeutic exercise.

Rehabilitation robotics has a long story starting in the early 1990s and an exhaustive reading is found in [2]. A robot in its simpler conception is a tool that consists of sensors, actuators, and a control system. The presence of force sensors allows an interactive control of the instrument measuring the forces exchanged with the subject. This also means it’s possible to study the characteristics of the human–machine interaction, a very current and widely studied topic that is gaining more and more attention in the field of robotic rehabilitation. The most distinctive feature of the devices used in rehabilitation is not their ability to “automate” treatment but the opportunity to precisely quantify sensory-motor performance during exercise in terms of movement kinematics and exchanged forces. That is, measuring to control the therapy and measuring for characterizing motor behaviors. The current generation of rehabilitative robots is designed to complement, rather than substitute, the therapist’s work.

Postural control studies date back to the beginning of the 20th century [3,4]. The evaluation of the human postural control system in dynamic conditions is generally performed by means of external body perturbations induced by movable platforms. Moving platforms were introduced in the field of the study of posturography in the 1970’s but since then, there has been no widespread dissemination. Examples of robotic mobile platform controlled in position or in impedance are presented in [5,6,7]. The most popular commercial apparatus for balance studies is the computerized dynamic posturography system, Equitest Neurocom (Otometrics, Natus Medical Srl, Montegrotto Terme, Italy) [8]. Recently, more complex devices and protocols were proposed [9,10,11,12] in order to evaluate the subject’s reaction to multiaxial perturbations but all of them with a maximum of 4 Degrees of Freedom (DoFs). The most diffuse commercial robotic platform with 6-DoF is CAREN (MOTEK Medical) [13]. It is a Stewart platform synchronized with a full virtual reality environment (VR). However, commercial platforms have two limits that have acted against the diffusion: they are sold with limited and pre-configured solutions and/or they are expensive.

To overcome the above-mentioned limits, we propose an ad hoc robotic platform: DORIS (Dynamic Oriented Rehabilitative Integrated System). The first aim of this project is to realize a versatile solution that can be applied both for research purposes but also for personalizing the training of the equilibrium and gait. The second one is to realize a solution that can be integrated with motion analysis systems, virtual reality and other robotic devices and sensors (including exoskeletons). This platform can accomplish any arbitrary movement and such a device could be effectively used in different fields, not only as a motion simulator for biomechanical evaluations but also for virtual reality applications mainly oriented towards sensorial manipulation. The intent is also to enhance the interactive characteristics of the platform, providing specific reactive poses of the platform and feedback to the subject, in accordance with the personal motor abilities.

The main aim of this paper is to introduce DORIS to the scientific community providing an exhaustive description of the mechanics, the controls and the integration with other systems in order to guarantee smarter and more efficient physical rehabilitation services. The techniques used to design and develop the manipulator and its controls have been largely studied and we briefly review them focusing on our conditions and needs. As a new inherent advantage, the integrated multi-system DORIS brings the possibility to gain a complete vision of the patient behavior and a complete control on the possible stimuli one could interact with the sensory-motor system of the person, by using the motion capture system, the electromyography system, and the virtual reality environment. This intelligent multi-system is something new in our knowledge and pave the way to gain novel insights about human sensory-motor framework and physical rehabilitation concepts.

## 2. Materials and Methods

### 2.1. Description of the Robotic Platform

**Figure 1:**
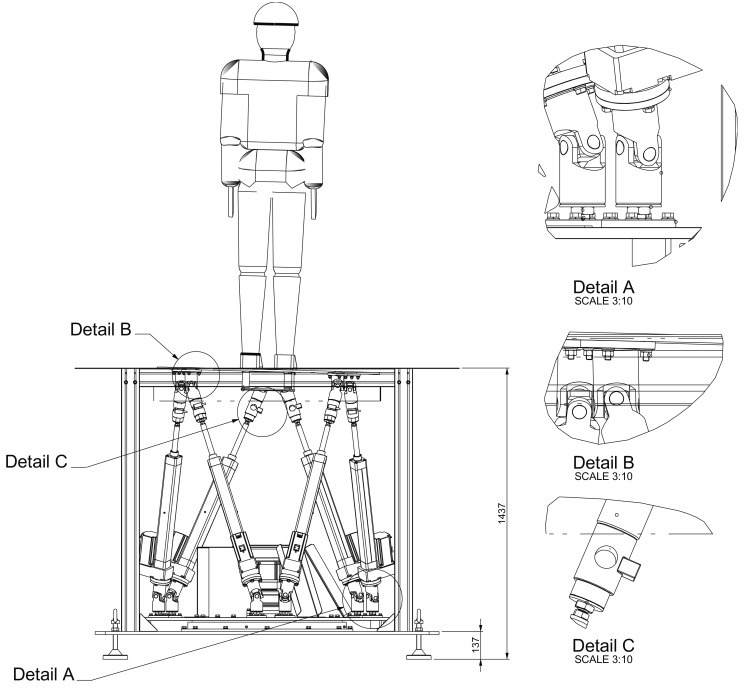
Mechanical design of the platform. Details A and B show the cardan joints that attached each leg with the two platforms. Detail C shows the amplified load cell mounted on each leg.

The robot we developed is a custom Stewart Platform mechanism. A standard Stewart platform is a parallel manipulator, it was first suggested by Gough and then presented to academia in 1965 by Stewart [14]. Stewart platforms have found their application in a variety of scenarios, such as, flight simulators, parallel robot manipulators, machine tools, 6 DoFs coordinate measuring devices, entertainment and health equipment. Stewart platforms inherent advantages over the conventional serial mechanism are simpler structure, higher stiffness, better accuracy, and heavier loading capabilities. It mainly consists of a lower base plate, an upper mobile plate (DORIS: Ø 1100 mm) and six identical stretchable legs (see Figure 1). Each end of the legs is attached to each plate with a Cardan joint (see Figure 1 Details A and B).

Each leg is actuated by a linear actuator (Rexroth Bosh group, Lohr a. Main, Germany). The features of the motors we adopted are summarized in Table 1.

In our DORIS system, we mounted the Stewart platform on an actuated rotating support for enhancing the limited yaw range, which is usually bounded in a standard Stewart platform (1S-series OMRON Corporation, Kyoto, Japan). On the passive legs below the mobile plate, immediately after the cardan joints, we inserted amplified load cells (TCE-amp AEP Transducer, Cognento, Italy) whose nominal load has been specially lowered to 100 kg, see Figure 1 Detail C. In order to render the system more comfortable for our patients, the DORIS platform was mounted so that the mobile plate is at the floor level when at rest. The DORIS platform is currently available in the Movement Analysis Laboratory (MARlab) at the Children Hospital Bambino Gesù. The mobile plate is elevated from the ground only when a translation on the horizontal plane is necessary, hence avoiding impacts with the adjacent floor.

The robotic platform can be controlled both in position and in force. In fact, it is possible to generate trajectories and/or control the platform behavior as a function of external feedback signals, such as the platform torque computed by the outputs of the load cells. The robot is controlled by a motion control and acquisition system based on a Computer Numerical Control (CNC) machine (NGWarp, Promax, Castelfiorentino, Italy).

### 2.2. System Architecture

**Figure 2:**
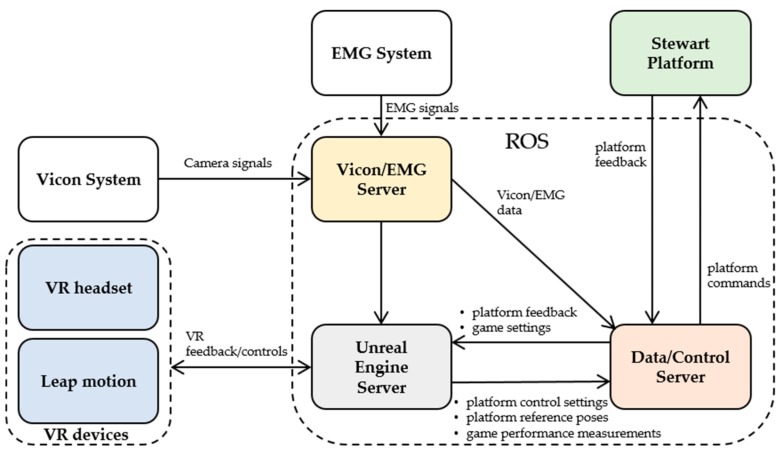
The system functional architecture of DORIS. The right dashed box includes all the main modules which use ROS (Robotic Operating System) as middleware. These subsystems exchange different kind of messages, whose main conveyed information is concisely reported over the arrows.

A functional diagram of the DORIS system is shown in Figure 2. The main system modules of DORIS are listed below.

• The Data/Control server, which is in charge of (i) collecting, routing and recording all the main data flowing in the system, (ii) controlling the Stewart platform and (iii) visualizing and monitor the state of the Stewart platform.
• The Stewart Platform, which has been presented in the previous section.
• The Unreal Engine server, which hosts Unreal Engine 4, the adopted game engine. This is responsible for generating the realistic VR game experiences which are used for assessing the neuro-rehabilitation patients’ capabilities and extrapolate relevant performance measures.
• The VR devices, i.e., the control interfaces used for the VR game experiences. These consist of a VR headset (an Oculus Rift S headset) and a Leap motion module (used for detecting and tracking hand gestures).
• The Vicon system, which is composed of a set of 12 cameras and variously deployed markers used to perform accurate motion capture of patients’ and system assets.
• The Electromyography (EMG) system, used for measuring and recording the electrical activity produced by the skeletal muscles of the patients.
• The Vicon/EMG server, which is in charge of (i) collecting, synchronizing and processing all the signals and data coming from both the Vicon system and the EMG system, and (ii) make all the processed sensory data available to the other DORIS subsystems.

Further details about the integration of the different DORIS modules, the data flows, and the software design are provided in Section 3.

### 2.3. Inverse Kinematic

**Figure 3:**
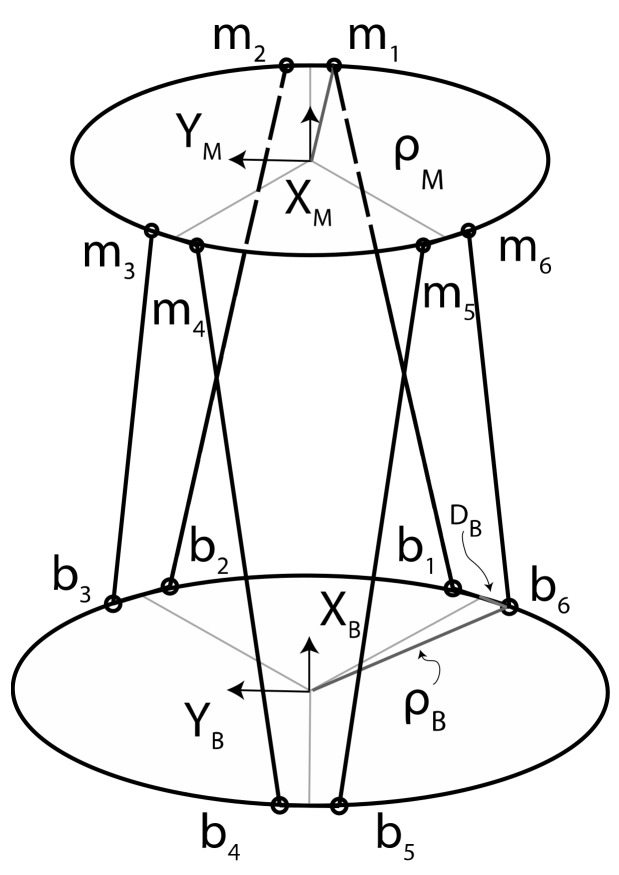
Platform geometric scheme with frame assignments and connecting points. The frame {B} is the reference frame assigned to the base plate, while the frame {M} is attached to the moving plate.

The direct kinematics of the Stewart platform has drawn the attention of many researchers and its resolution is still under investigation, as it has not yet been completely solved in an efficient and robust way. Several authors propose solutions for this problem [15,16,17,18,19,20]. On the other hand, solving the problem of the inverse kinematics, i.e., determining the leg lengths once the position and orientation of the mobile plate are known, is easy to achieve. In a general robotic system, inverse kinematics deals with the determination of a set of joint variables, which yield a set of variables that define the *workspace*, usually consisting of 3D position and orientation of the robot end-effector with respect to a selected reference frame [21]. In our case, the workspace could be also characterized by the 3D coordinates of the six conjunction points of the legs with the two plates. In order to characterize and model the platform, we proceed by assigning two coordinate frames {B} and {M} to the base and the moving platforms respectively. As Figure 3 illustrates, the origin of frame {B} is the centroid B of the base plate, while the origin of frame {M} is the centroid M of the moving plate. When the platform is at rest, the Z axes of both frames point upward, while the X and Y axes lie on the horizontal plane.

Points b_i_ and m_i_ are the connecting points of the legs which connect the base to the mobile plate. We select as workspace variables the relative 3D position and orientation of frame {M} with respect to frame {B}. The connecting points of the base platform **b_1_** and **b_6_**, **b_2_** and **b_3_**, and **b_4_** and **b_5_** can be considered in pairs and the orientation of each couple is defined in the frame {B} by the vector $c_{B}\;=\;\left[-\pi /3,\;\pi /3,\;\pi \right]$. Therefore, the locations of the connecting points of the base platform for each of the previous pairs are

(1) $$b_{i}=\;\left[\begin{array}{c} b_{\mathrm{xi}}\\ b_{\mathrm{yi}}\\ b_{\mathrm{zi}} \end{array}\right]=\left[\begin{array}{c} \varrho_{B}\cos\left(\delta_{i}\right)\\ \varrho_{B}\sin\left(\delta_{i}\right)\\ 0 \end{array}\right]\;i\;=\;1,\;2,...,6\;$$

where 6 is the number of joints points $\delta_{2j-1}=c_{B}\left(j\right)+{\;\theta }_{B}$ and $\delta_{2j}=c_{B}\left(j\right)-\theta_{B}$, with j = 1, 2, 3 and $\theta_{B}=\mathrm{acos}\left(\frac{\sqrt{R_{B}^{2}-D_{B}^{2}}}{R_{B}}\right)$.

Considering that the mobile plate has a yaw offset of π/3, it is easy to express in the same way the **m_i_** joints points coordinates as

(2) $$m_{i}=\;\left[\begin{array}{c} m_{\mathrm{xi}}\\ m_{\mathrm{yi}}\\ m_{\mathrm{zi}} \end{array}\right]=\left[\begin{array}{c} \varrho_{M}\cos\left(\mu_{i}\right)\\ \varrho_{M}\sin\left(\mu_{i}\right)\\ 0 \end{array}\right]\;i\;=\;1,\;2,\;...,\;6$$

The pose of the moving plate with respect to the fixed frame {B} can also be expressed by the position vector **^B^d** and the rotation matrix **^B^R_M_** $\in \mathrm{SO}\left(3\right)$, see Equation (3)

(3) R BM=r11r12r13r21r22r23r31r32r33

In this study, the matrix R BM is defined by the roll  α, pitch β  and yaw γ  angles following the ZYX sequence.

Considering the vector **l_i_** = [l_ix_, l_iy_, l_iz_]^T^ of the i-th actuator, we express it with respect to the frame {B} as

(4) l Bi=m Bi−b Bi

Given **^B^d** = [x, y, z]^T^, the vector containing the 3D position of the origin of the frame {M} with respect to the frame {B}, and given the orientation matrix **^B^R_M_**, **m_i_** is obtained as

(5) m Bi= R BMm Mi+d B.

The inverse kinematic solution is then the norm of the vector **l_i_** of the link i-th,

(6) l Bi=R BMm Mi+d B−b Bi

A rotation matrix is normalized and orthogonal, and assuming that b_zi_ = m_zi_ = 0 and that $∥b_{i}∥=\varrho_{B}^{2}\;$ and $∥m_{i}∥=\varrho_{M}^{2}$, thus the length of vector **l_i_** is

(7) $$l_{i}^{2}=x^{2}+y^{2}+z^{2}+\varrho_{M}^{2}+\varrho_{B}^{2}+2\left(r_{11}m_{\mathrm{ix}}+r_{12}m_{\mathrm{iy}}\right)\left(x-b_{\mathrm{ix}}\right)+2\left(r_{21}m_{\mathrm{ix}}+r_{22}m_{\mathrm{iy}}\right)\;\left(y-b_{\mathrm{iy}}\right)+2\left(r_{31}m_{\mathrm{ix}}+r_{32}m_{\mathrm{iy}}\right)z-2xb_{\mathrm{ix}},\;\mathrm{for}\;i\;=\;1,\;2,\;\ldots ,\;6$$

Thanks to Equation (7) and given a desired platform configuration in the workspace, which is defined by its position **^B^d** and the orientation **^B^R_M_**, we can design a position control for the DORIS platform.

### 2.4. Force-Torque and Center of Pressure Sensor

One of the main goals in posturography is computing the center of pressure (COP). That is, the X and Y coordinates of the point of application of an external force ($F_{e}$) acting on the surface of the mobile plate. It is possible to compute the COP by using the statics (principle of virtual work). In particular, this can be achieved by measuring the three components of the force applied to the platform $F_{e}=\left(F_{X},{\;F}_{Y},{\;F}_{Z}\right)$ and of the moment of the force $T_{e}=\left(T_{X},\;T_{Y},\;T_{Z}\right)$, referred to the origin of the frame M. Measuring external forces and torques means to consider the Stewart platform as a force-torque sensor, as proposed in past studies [22,23]. The force and the moment acting on the platform are distributed on the six legs. Therefore, by introducing a transformation matrix **H**, it is possible to relate the forces $f_{i}\;$ measured by each leg with the external force and moment ($F_{e},\;T_{e}$). The formulation of the **H** matrix is quite simple. Being ${\hat{l}}_{i}$ the unit vector of l Bi, the leg force is given by ${\hat{l}}_{i}f_{i}$ and the three components of the external force and of the moment, at the equilibrium, can be computed by the following set of equations

(8) $$\left\{\begin{array}{c} \sum_{i=1}^{6}{\hat{l}}_{i}f_{i}=\;F_{e}\\ \sum_{i=1}^{6}b_{i}\times \;{\hat{l}}_{i}f_{i}=\;T_{e} \end{array}with\;i=1,\;2,..,\;6;\right.$$

that expressed in matrix form allows to identify the 6 × 6 transformation matrix **H**:

(9) $$\left[\begin{array}{ccc} {\hat{l}}_{1} & \ldots & {\hat{l}}_{6}\\ b_{1}\times {\hat{l}}_{1} & \ldots & b_{6}\times {\hat{l}}_{6} \end{array}\right]\left[\begin{array}{c} f_{1}\\ \vdots \\ f_{6} \end{array}\right]=\left[\begin{array}{c} F_{e}\\ T_{e} \end{array}\right]$$

Indeed, the matrix **H** is the inverse transpose of the geometric Jacobian matrix, which is used in the differential kinematics equation [24]. The matrix **H** is singular when the forces distributed on the legs are linearly independent. This entails that some components of external forces and moments cannot be supported by the platform, and if the platform is near a singular configuration small values along the singular direction lead to large axial forces read by the load cells [23].

Given the components of both the force and the moment it is possible to compute the COP coordinates [25]:

(10) $$\begin{array}{c} X_{COP}=\;-\frac{{T^{\prime }}_{Y}}{F_{Z}}\\ Y_{COP}=\;\frac{{T^{\prime }}_{X}}{F_{Z}} \end{array}$$

where ${T^{\prime }}_{X}$ and ${T^{\prime }}_{Y}$ are the components of the moment acting on the top plate, expressed with respect to the frame {M}. This approach stands on the assumption that the top plate can be considered as an infinitely rigid body. Deformations of the plate introduce errors. In general, non-linearities in the mechanical structure, electronic noise, hysteresis and different offset voltages can be sources of error. In order to avoid these sources of error and to obtain measures as accurate and precise as possible, appropriate calibration and quality control tests are suggested [26,27]. Several authors proposed to quantify force plates output errors by comparing the COP coordinates measured by the platform with the coordinates of a reference COP obtained from a motion capture system. In particular, a reference COP can be computed by acquiring the position of markers located on a rigid rod, which is instrumented with a load cell while forces with different directions and locations are applied on the platform [28,29,30]. In particular, Collins et al. [27] described a very useful procedure, named Post-Installation Least-Square Procedure (PILS) that aims at correcting the errors introduced during the installation of the platform.

We followed this procedure by applying arbitrary forces to the mobile plate of DORIS with the modified pole MTD-2 (Motion Lab Systems, Baton Rouge, LA, USA). This pole was instrumented with a single axis load cell (LCM200, FUTEK Advanced Sensor Technology, Inc., Irvine, CA, USA) near the point of contact with the platform allowing the measure of the magnitude of a reference force. The signal from the load cell was integrated into the motion capture system used in the MARlab (12 cameras Vicon Vero, Oxford Metrics, Oxfordshire, UK) that can accept analogical input at a frequency of 1000 Hz. We acquired rod markers positions with the motion capture system with a sample rate of 200 Hz. These markers provide the possibility to find the direction of the reference force applied to the platform through the pole, and to consequently compute the reference torque too. The calibration procedure consisted of the acquisition of 20 trials each one lasting approximately 10s and in different locations. Loading was provided by the body weight pushing on the pole while it was slowly tilted about the contact point. Therefore, the load-cell output and the pole markers positions define the reference matrix **R**. In particular, each column of **R** represents the six force and moment components along the three axes of the laboratory coordinate system. While the signals measured by the Stewart platform define the matrix **S**. Their detailed computations are described by Collins et al. [27]. Given the matrices **R** and **S**, it is possible to compute the calibration matrix **C**. The sampling rate of DORIS is 250 Hz and the two signals were synchronized with respect to the first force peak. The matrix **C** is used to correct the matrix **S**. Indeed, the matrix **C** plays a crucial role in posturography for measuring the COP and other related measurements.

## 3. Architecture Details

In this Section, we provide further details about the integration of the different DORIS subsystems and its software design.

### 3.1. System Modules and Data Flow

**Figure 4:**
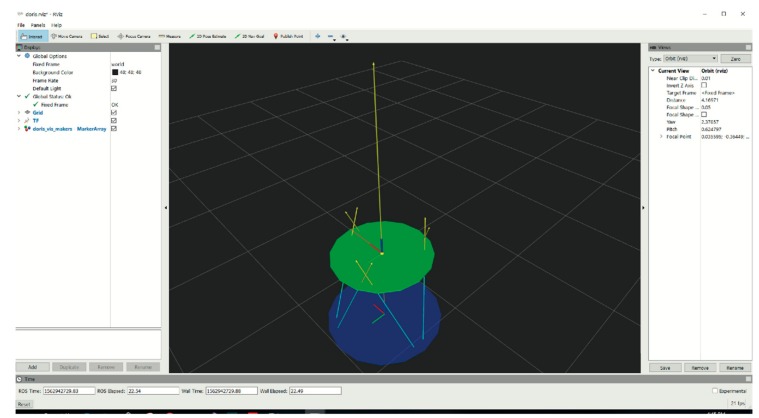
The Graphical User Interface (GUI) implemented with the ROS RVIZ interface. At all times, the state of the Stewart platform, with its reaction forces and the center of pressure (COP), can be visualized and monitored on this GUI.

An overview of the different subsystems is provided in Section 2.2. As reported in Figure 2, the main modules exchange different kinds of messages by using the Robot Operating System (ROS) as middleware [31]. In particular, all the acquired sensory data are re-emitted in real-time over ROS topics by using the convenient ROS publisher-subscriber paradigm. The implemented messages convey sensory data, synchronization/coordination information, configuration parameters and load/save commands. Notably, the use of ROS allows to easily record the history of all the data messages (including Vicon and EMG data, platform feedback and commands) which normally flow over the network during each working session. The recorded rosbag files contain interlaced, serialized ROS messages along with their global timestamps, dumped directly into a single log file as they come over the network wire. This allows to play-back the recorded messages in a time-synchronized fashion and to further post-process the data of interest. In particular, within our framework, the state of the Stewart platform, with its reaction forces and the COP, can be conveniently visualized and monitored by using the ROS RVIZ (ROS visualization) interface, as shown in Figure 4.

### 3.2. Sofware Design

**Figure 5:**
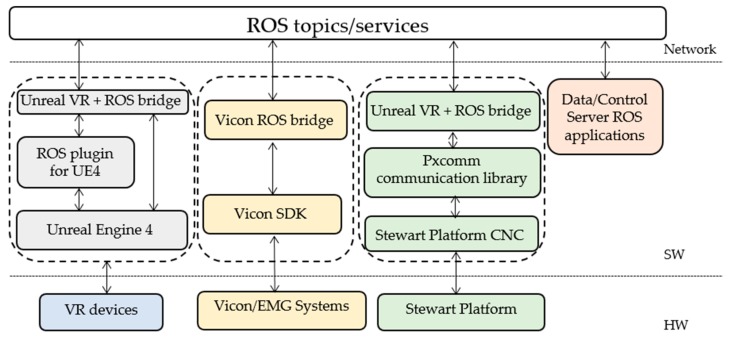
The software architecture of DORIS. The hardware (HW), software (SW) and network layers are sketched (separated by blue dashed lines). Each solid box represents a different subsystem or software layer. Each stack (dashed box) represents a different software module with its stratification.

Figure 5 shows a sketch of the software layers and interfaces (depicted as solid boxes). The main hardware (HW), software (SW) and network layers are sketched within the same figure and separated by dashed lines. Each stack (dashed box) represents a different software module with its stratification and constituent software layers. As detailed above, all the software modules are provided with ROS interfaces, also referred to as *ROS bridges*.

The Stewart platform stack (in green), includes the following components.

• The CNC module (programmed both in Visual Basic and C), which is hosted in the Stewart platform firmware.
• The Pxcomm communication library (programmed in C/C++), which allows the communication with the CNC module through the TCP/IP protocol. Specifically, this library has been designed to allow the integration of the Stewart platform with other systems.

The Vicon ROS bridge is built on the top of the Vicon SDK and publishes synchronized Vicon and EMG data towards the other ROS applications.

The Unreal Engine stack uses an open-source plugin, which is named ROSIntegration (https://github.com/code-iai/ROSIntegration), which allows to add ROS support to our Unreal Engine project. In this regard, we developed a few game experiences for virtual reality (Unreal VR) by replacing the standard VR hand-controllers with a Leap Motion controller, which can detect and track hand gestures in order to let the patient more comfortably interact with the VR scenarios. Notably, in the DORIS framework, both the Stewart platform feedback and the Vicon marker measurements can be additionally used for VR interaction (e.g., the Stewart platform can be used as a compliant surfboard by the patient). The Data/Control server can host many other ROS-based applications for different purposes such as visualization/monitoring (e.g., the RVIZ GUI shown in Figure 4), data recording and post-processing.

Indeed, the DORIS software was designed with the main goals of allowing (i) an easy integration with new systems and (ii) a flexible development of personalized training exercises and experimental protocols.

## 4. Results

### 4.1. Movement Assessment of the Platform

The motion accuracy of the DORIS platform in replicating a chosen pose was performed comparing the data obtained from the DORIS leg extensions (obtained by the inverse kinematic) **l_i_** with the data collected by the motion capture system installed in the MARlab. Ten markers were placed on the mobile plate in order to easily reconstruct the locations of the connecting points **m_i_** with respect to the laboratory frame {L}, **^L^mv_i_**. We executed two tests to evaluate the accuracy of movement. In the first test, we imposed a translation on the vertical axis of 200 mm. In the second test, we applied a sinusoidal rotation about the x-axis of the platform (roll, with amplitude A = 5° and angular velocity ω = 10 rad/min, i.e., $roll=A\cdot \sin\left(\omega t\right)$). By using the transformation matrix **^B^R_L_** we obtained the **^L^mv_i_** with respect to frame {B} of the Vicon data, i.e., m Bvi=R BLm Lvi. Given the history of the platform poses, we then simulated the whole control of the platform in Matlab (Mathworks, Natick, MA, USA) and computed the **^B^ms_i_** points with respect to the base frame. We assessed the accuracy of each test by computing the Root Mean Square Error (RMSE) of each component of the six points **m_i_**. In particular, we compared the values computed starting from motion capture system with the values computed starting from the platform measurements.

**Figure 6:**
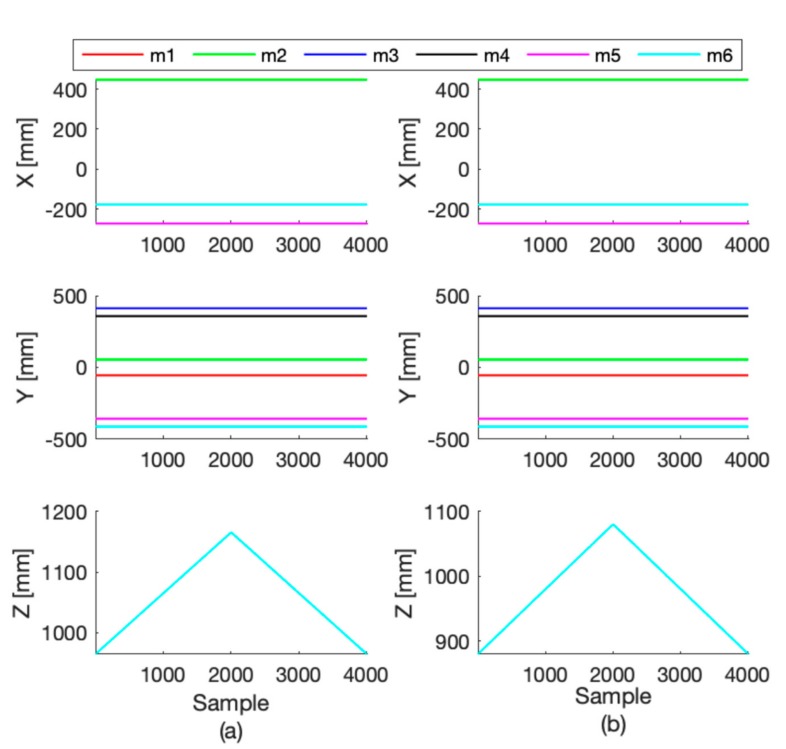
Cartesian coordinates of the connecting points of the mobile plate m_i_ (one color each point) while it is elevating from the floor up to 200 mm (first test). (**a**) Data captured by the cameras system. (**b**) Platform data.

**Figure 7:**
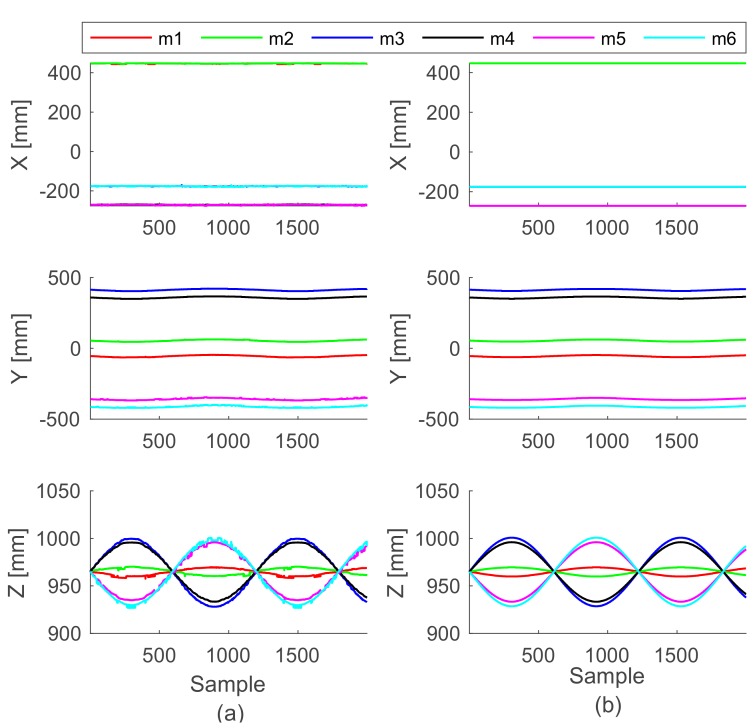
Cartesian coordinates of the connecting points of the mobile plate m_i_ (one color each point) while the roll angle follows a sinusoidal time law. (**a**) Data captured by the cameras system. (**b**) Data reproduced by the simulation algorithm.

Figure 6 and Figure 7 show the Cartesian coordinates of each connecting point of the mobile platform (one color for each point m_i_) while performing the two tests. RMSE values of both tests (along with their standard deviations) are shown in Table 2 and Table 3. Notably, during the first test (while the platform was simply elevated from the floor), the RMSE values of all the X and Y coordinates remained 0.8 mm, exception made for the connecting points of the mobile plate m_5_ and m_6_ that are maximum 2.4 mm. The Z coordinate shows an error that goes from 1.5 to 2.3 mm. While the plate was moving according to the sinusoidal time law, the RMSE was always bigger along the Z axis, but in general increased in all axes. In order to evaluate the repeatability of the platform behavior, we also acquired three consecutive movements of the first test, and we found that the RMSE between these repetitions was 1.44 ± 0.9 mm. 

### 4.2. Center of Pressure Measurement Assessment

**Figure 8:**
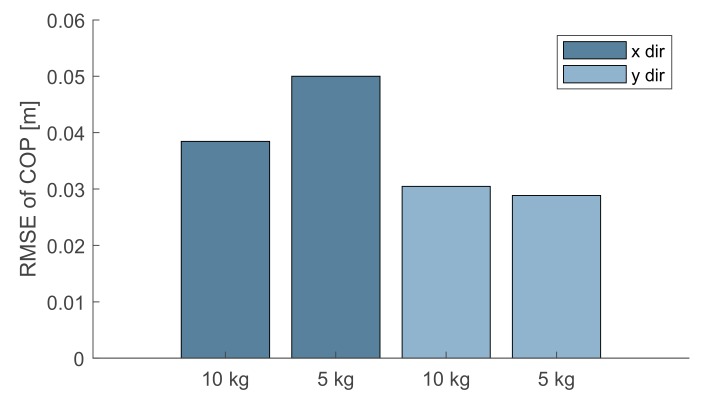
RMSE between $COP_{Calib}\;$

The correction of errors while measuring forces, moments and COPs was performed following the four steps of the PILS procedure [27]. In order to test the accuracy of the corrected output we performed 10 trials—each one lasting 5 s. In each of these acquisitions, we computed the COP coordinates *COP_Calib_* starting from the signal of the loading cells with the mobile platform loaded with calibrated loads of 10 kg and 5 kg, which were positioned at 10 cm from the platform center. Each test was repeated 5 times. In particular, we used the calibration matrix **C** given by the PILS procedure and we compared the COP values by using the RMSE of X and Y directions with the gold standard ($COP_{G}$). $COP_{G}\;$ was estimated by the markers located on the loads and tracked by the motion capture system and expressed with respect to the platform base frame {B}. Figure 8 shows the RMSE between $COP_{Calib}\;$ and $COP_{G}.\;$ COP measurements revealed a RMSE between 0.04 and 0.05 m along the X axis and of 0.03 m along the Y axis and $COP_{G}$. Darker grey bars denote the RMSE in the X direction. Light grey is the RMSE in the Y direction.

**Figure 9:**
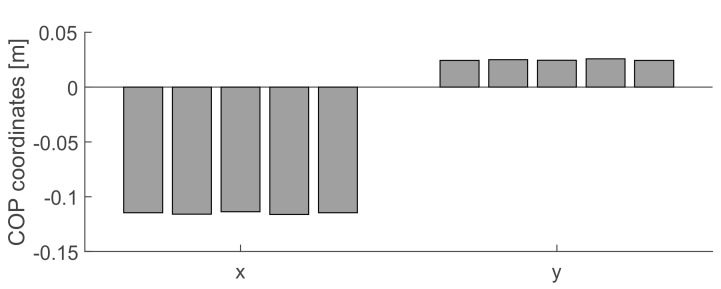
Stability test. Each bar is the component X and Y of the $COP_{Calib}$ computed at time intervals of 1 minute.

In order to evaluate possible drifts and temporal stability, we acquired over 5 minutes the calibrated load of 10 kg and then compared calibrated $COP_{Calib}\;$ coordinates at time intervals of one minute. Figure 9 shows the very little drift we observed for the COP coordinates. The variance between the five measures of the X coordinate is 1.13 μm and of the Y is 0.43 μm.

## 5. Discussion

This paper is the first introduction of DORIS to the scientific community. We first provide an exhaustive description of its design, mechanics, the controls. Additionally, we present how we integrated this robotic platform with other systems in order to guarantee a smarter and a more versatile use for research and physical rehabilitation services. We described the techniques we used to design and develop the robotic platform and its controls starting from the state of the art but focusing and aiming at our specific rehabilitation objectives.

In order to assess the platform motion accuracy and evaluate measurement repeatability, we performed different sensory acquisitions by using both the platform load cells and the Vicon system. Then, we processed and compared all the collected data offline. 

In a first test, with the mobile plate vertically elevating from the floor up to 200 mm, the RMSE shows an error almost constant and under 2.4 mm along both the X and Y axes. On the other hand, along the direction of movement, the RMSE increases up to a maximum of 2.3 mm. In a second test, with the mobile plate roll angle following the sinusoidal function, the RMSE stays under 1.3 mm on the X component. During the vertical elevation the observed points that are involved in the movement, show an increase of the RMSE in time up to a maximum of 1.7 mm along y-axis and of about 3.1 mm along the Z axis. In this regard, it is worth noting that the m_i_ connecting points are estimated from the mounting points of the legs under the mobile platform (Figure 1 Detail B). These computations come with some approximations which introduce errors and measurement uncertainty. The effects of these approximations become more evident at the level of the connecting point m_5_ and m_6_, see the RMSE values in Table 2. Acceptable repeatability of the platform behavior (RMSE 1.44 ± 0.9 mm) was found.

Moreover, another origin of measurement uncertainty is given by Vicon system whose accuracy is reported to be about 2 mm [32] in some works. Therefore, we expect to attain some improvements for the actual RMSE values in future works, by introducing more advanced modelling and estimation procedures for the connecting point **m_i_**.

As for the performed posturography tests, in the observed worst cases, COP measurements revealed a RMSE of 0.05 m along the X axis and of 0.03 m along the Y axis. As expected RMSE increased when the weight decreased. It is also possible to observe that our platform was more accurate along the Y axis. In the literature measurment error of standard platforms fabricated expressly for posturography are reported around 1 mm [26,27,33]. However, this error is also affected by positioning errors of the load of about 15 mm and by the other measurement errors introduced by the optoelectronic system while tracking both the platform and the loads. Considering that this platform will be used with pediatric patients with a minimum weight of 20 kg, we expect to attain a higher level of accuracy. Indeed, even with lower loads, the estimated RMSE values appear acceptable. No drift was found by the last test described. This is fundamental for long sessions of testing with patients. Even in this second test, the connecting points estimation errors are so contained that they could be regarded as originated by the Vicon measurement uncertainty.

These preliminary results show a level of accuracy greatly acceptable for use during human interaction. However, further experiments and analyses are required for reducing the range of uncertainty in the measurement chain, mainly by reducing the error in the estimation of the connecting points of the platform. Meanwhile, we expect that increasing the positions of the calibrated loads will conduct on a more precise map of DORIS accuracy.

## 6. Conclusions

In this work, we presented the DORIS platform. In order to evaluate its capabilities as a custom Stewart platform, we reviewed, described, and tested some fundamental techniques, from position control to force and torque measurements, in a variety of contexts but not in the rehabilitative field. To the best of our knowledge, no previous work describes in such a complete way a robotic instrument aimed at measuring postural stability and studying human motor control, with the ultimate goal of discovering new rehabilitative techniques. The authors hope that this work can be useful for future studies in the field of diagnostic and rehabilitation assessment through such a versatile technology. This intelligent and open (With this perspective a web site for describing the DORIS project and its work in progress was prepared at the following link: https://github.com/MARlab-opbg/DORIS) multi-system is something new in our knowledge and pave the way to deep insights about human sensory-motor framework and physical rehabilitation concepts.

## Figures and Tables

**Table 1:** Main features of the motors.

Electro-Mechanical Cylinder System—EMC-050NN-2	
Max. stroke	400 mm
Lead constant	20 mm/U
Max travel speed	1.27 m/s
Max acceleration	50 m/s^2^
Max drive torque	8.2 Nm
Gear reduction	1

**Table 2:** RMSE values during the first test (platform elevating along the Z axis) computed from data measured by DORIS platform and by the cameras system. Mean value for each axis ± standard error.

Connecting Point	X (mm)	Y (mm)	Z (mm)
m1	0.7 ± 0.008	0.7 ± 0.005	1.6 ± 0.01
m2	0.7 ± 0.006	0.8 ± 0.007	1.9 ± 0.01
m3	0.7 ± 0.007	0.3 ± 0.003	2.1 ± 0.02
m4	0.4 ± 0.005	0.6 ± 0.006	1.6 ± 0.01
m5	1.5 ± 0.01	2.2 ± 0.02	1.5 ± 0.01
m6	2.4 ± 0.02	1.2 ± 0.03	2.3 ± 0.02

**Table 3:** RMSE values during the second test (roll angle following a sinusoidal time law) computed from data measured by the DORIS platform and by the cameras system. Mean value for each axis ± standard error.

Connecting Points	X (mm)	Y (mm)	Z (mm)
m1	0.8 ± 0.02	1.1 ± 0.02	0.6 ± 0.01
m2	0.7 ± 0.01	1.1 ± 0.02	0.5 ± 0.009
m3	0.9 ± 0.02	1.0 ± 0.01	2.9 ± 0.05
m4	0.9 ± 0.02	0.9 ± 0.01	2.4 ± 0.04
m5	1.1 ± 0.02	1.7 ± 0.03	2.4 ± 0.04
m6	1.3 ± 0.03	1.7 ± 0.04	3.1 ± 0.05
